# Elevation of IL-8 secretion induced by PEDV infection via NF-κB signaling pathway

**DOI:** 10.3389/fcimb.2024.1422560

**Published:** 2024-07-22

**Authors:** Yang Wu, Yongrui Wang, Xuepeng Wang, Mingwei Li, Haoxin Yan, Hongyan Shi, Da Shi, Jianfei Chen, Longjun Guo, Li Feng

**Affiliations:** State Key Laboratory for Animal Disease Control and Prevention, Harbin Veterinary Research Institute, Chinese Academy of Agricultural Sciences, Harbin, China

**Keywords:** PEDV (porcine epidemic diarrhea virus), proinflammatory cytokine, interleukin 8, M protein, E protein

## Abstract

Porcine epidemic diarrhea virus (PEDV) is associated with severe enteritis, which contributes to high mortality in piglets. The aim of this study was to describe molecular mechanisms associated with proinflammatory cytokine(s) production during PEDV infection. We showed that infection of porcine intestine epithelial cell clone J2 (IPEC-J2) with PEDV induces a gradual increase in interleukin 8 (IL-8) production at different time points, as well as infection of Vero E6 with PEDV. The secretion of IL-8 in these two cell lines infected with PEDV is related to the activation of NF-κB. Furthermore, the cells expressing PEDV M or E protein can induce the upregulation of IL-8. These findings suggest that the IL-8 production can be the initiator of inflammatory response by the host cells upon PEDV infection.

## 1 Introduction

Porcine epidemic diarrhea virus (PEDV) is an enveloped virus with a positive-stranded RNA, belonging to the genus *Alphacoronavirus* within the family *Coronaviridae* (Perlman and Netland, 2009). The genome of PEDV is comprised of at least seven open reading frames that encode three non-structural proteins (replicases 1a/1b and ORF3) and four structural proteins which are arranged on the genome in the order 5’-replicase (1a/1b)-S-ORF3-E-M-N-3’ (Egberink et al., 1988; Bridgen et al., 1998). The four major structural proteins are located at 3’-terminal end of the genome: spike (S), envelope (E), membrane (M) and nucleocapsid (N) (Kocherhans et al., 2001; Chen et al., 2011).

PEDV has been identified as the etiologic agent of porcine epidemic diarrhea (PED), and causing a mortality rate of 80–100% in suckling piglets (Song and Park, 2012; Sun et al., 2012; Chen et al., 2014). Despite the availability of vaccines, the PED has broken out frequently in many swine-raising countries, resulting in considerable economic loss (Chen et al., 2013; Kim et al., 2015). Epidemic PEDV strains are highly enteropathogenic and mainly infect villous epithelial cells of the small intestine (Jung et al., 2014). One characterization of activation of epithelial cells is their production of proinflammatory cytokines, which act *in vivo* to attract immune cells to site of infection and initiate the inflammatory response (Kagnoff and Eckmann, 1997). Therefore monitoring of epithelial cells may provide insight into the inflammation process. However, how PEDV infection affects inflammatory response is little known.

The innate immune system serves as the first line of defense against invading pathogens. Coronaviruses are recognized by cytosolic and/or endosomal pattern recognition receptors (PRRs), which activate adaptor proteins and downstream pathways. This eventually leads to the activation of critical transcription factors such as nuclear factor kappa light chain enhancer of activated B cells (NF-κB), interferon regulatory factor 3/7 (IRF-3/7), and activator protein 1 (AP-1). These proteins then activate the transcription of type I/III interferons (IFN-I/III) and proinflammatory cytokines/chemokines such as tumor necrosis factoralpha (TNF-α), interleukin-6 (IL-6), and IL-8 (Cheong et al., 2023). Acting locally, these cytokines/chemokines recruit immune cells and facilitate antiviral responses; but their excessive and uncontrolled release can lead to life-threatening cytokine release syndrome (CRS) that underlies the pathogenesis of severe coronavirus diseases (Chua et al., 2020; Sallenave and Guillot, 2020; Gustine and Jones, 2021). However, it is still unclear whether acute PEDV infection induces pro-inflammatory cytokine immune responses.

In the present study, we demonstrated that PEDV infection induced IL-8 upregulation. We then demonstrated that the secretion of IL-8 is related to the activation of NF-κB. Subsequently, we showed here that PEDV E and M protein induced IL-8 production. Our study provides further information for our better understanding of PEDV pathogenesis.

## 2 Materials and methods

### 2.1 Cell culture and viruses

Porcine small intestine epithelial cell clone J2 (IPEC-J2; ATCC) and African green monkey kidney cell line, clone E6 (Vero E6; ATCC) were cultured in Dulbecco’s minimum essential medium (DMEM) (Life Technologies, USA) supplemented with 10% heat-inactivated fetal bovine serum (FBS) (Gibco, USA), 100 U/ml penicillin, 100 μg/ml streptomycin at 37°C in an incubator with 5% CO_2_ at 37°C (Thermo Scientific, USA). PEDV strain (GenBank accession number KT323979) was prepared and titrated as previously described (Yang et al., 2018).

### 2.2 Primers and antibodies

The recombinant pCAGGS plasmids containing individual PEDV viral protein (E, M and N) with a Flag fusion tag and IL-8 promoter plasmid were kept in our lab. The listed antibodies were used in this study including anti-Flag mouse monoclonal antibody (mAb) and anti-β-actin primary antibodies (Cell Signaling Technology, USA). IRDye-conjugated secondary antibodies were purchased from Li-Cor Biosciences.

### 2.3 Virus infection and drug treatments

Monolayers of IPEC-J2 or Vero E6 cells were infected with PEDV at an MOI of 1 for 1 h at 37°C. Unbound virus was removed, and cells were maintained in complete medium for various time points until samples were harvested. For some experiments, different treatments with NF-κB inhibitor Bay 11–7082 (Sigma), or carrier DMSO (Sigma) were performed in the target cells as detailed in the corresponding Figure Legends.

### 2.4 Transfection

Cells were transfected with indicated plasmids using X-tremeGENE transfection reagent according to manufacturer’s instruction (Roche, USA). At the indicated times, cell samples were collected and lysed in RIPA buffer (Beyotime, Nantong, China) for western blot analysis of target proteins.

### 2.5 Western blot

Western blot analysis was performed as previously described (Luo et al., 2017). Treated samples were lysed in RIPA buffer containing protease inhibitor cocktail and phosphatase inhibitors (Roche, Switzerland) and separated by SDS-PAGE under reducing conditions and transferred onto a PVDF membrane (Merck Millipore, USA). After blocking, the membranes were incubated with a primary antibody and then probed with an appropriate IRDye-conjugated secondary antibody (Li-Cor Biosciences, Lincoln, NE). The membranes were scanned using an Odyssey instrument (Li-Cor Biosciences) according to the manufacturer’s instructions.

### 2.6 Quantitative RT-PCR

Quantitative RT-PCR (qRT-PCR) analysis was carried out as described previously (Guo et al., 2014). Total RNA was extracted from cells and subjected to qRT-PCR using specific primers as listed in **Table 1**. Relative gene quantification was performed by the method of 2(-Delta Delta C(T)) (Livak and Schmittgen, 2001).

**Table 1:** Primers used in this study.

Primer	Forward (5'→3')	Reverse (5'→3')	Usage
qIL-8	CCACACCTTTCCAC CCCAAA	TTGTTGCTTCTCA GTTCTCTTCA	Quantitative RT-PCR
qTNF-α	GCGTGGAGCTGAGA GATAAC	ATAGTCGGGCCGAT TGATCT	Quantitative RT-PCR
qIL-12	AAACCAGCACAG TAGAGG	TTGTGGCACAGT CTCACT	Quantitative RT-PCR
qIL-18	ATTGACAGTACG CTTTAC	GATGTTATCAGG AGGATT	Quantitative RT-PCR
q β-actin	CTTCCTGGGCATG GAGTCC	GGCGCGATGATCT TGATCTTC	Quantitative RT-PCR

### 2.7 Dual luciferase reporter assay

Vero E6 cells were co-transfected with pNF-κB-Luc, pRL-TK (Promega Biotech Co., Ltd, Beijing, China) by using X-tremeGENE reagents (Roche, USA). At 24 h post-transfection, the cells were infected with PEDV. Then, the cells lysates were prepared, and dual luciferase reporter assays were carried out in a GloMax 96 microplate luminometer (Promega, Beijing, China) using a Dual-Gloluciferase kit (Promega, Beijing, China) according to the manufacturer’s instructions.

Vero E6 cells were co-transfected with porcine IL-8 promoter vector, pRL-TK and pCAGGS/E/M/N-Flag by using X-tremeGENE reagents. Meanwhile, porcine IL-8 promoter vector, pRL-TK, and pCAGGS-Flag were also co-transfected as a control group. At 30 h post-transfection, the dual luciferase reporter assays were performed.

### 2.8 ELISA

The IL-8 protein levels in plasma and cell culture supernatants were measured using porcine IL-8 ELISA kits (RD Systems) in accordance with the manufacturer’s instructions.

### 2.9 Animal experiment

Four 5-day-old specific-pathogen-free (SPF) pigs were randomly assigned to 2 experimental groups: The infected group 1 (n = 2) and the uninfected group 2 (n = 2). The two different groups were challenged orally with PEDV CV777 (2 ml of 10 ^6.0^ TCID_50_/mL), and DMEM served as the mock control. All the pigs were euthanized for pathological examination when the onset of clinical signs was observed. Fresh samples, including colon, duodenum, jejunum and ileum, were collected during the necropsy. The fresh samples and piglets serum were collected for IL-8 detection.

### 2.10 Statistical analysis

All statistical analyses were performed using GraphPad Prism 9 (GraphPad Software, Inc.). Variables are expressed as mean ±S.D. Statistical analyzes were performed using student’s t test. A *P* value of <0.05 was considered significant.

## 3 Results

### 3.1 PEDV induces IL-8 production *in vitro*

**Figure 1:**
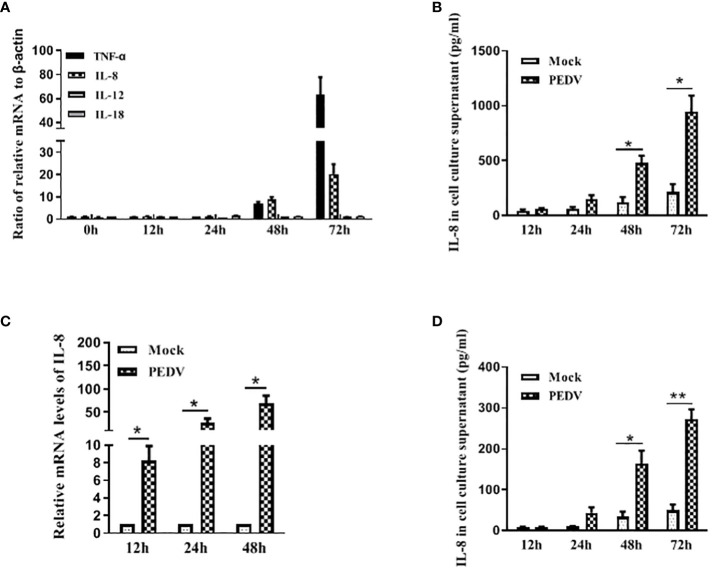
PEDV infection induces IL-8 upregulation *in vitro*. **(A, B)** PEDV infection induces IL-8 upregulation in IPEC-J2 cells. IPEC-J2 cells were exposed to PEDV CV777 at an MOI of 1 or mock control for different time points as indicated. The mRNA levels of TNF-α, IL-8, IL-12 and IL-18 were quantified by qRT-PCR using the primers specific to the target genes **(A)**. Supernatants were collected from the cells infected with PEDV CV777 or mock control, and followed by ELISA analysis to examine the IL-8 protein level **(B)**. **(C, D)** PEDV infection induces IL-8 upregulation in Vero E6 cells. Vero E6 cells were infected with PEDV CV777 at an MOI of 1 or mock control for different time points as indicates. The levels of IL-8 were quantified by qRT-PCR **(C)**. Supernatants were collected from cells infected with PEDV CV777 or mock control, and were assessed by IL-8 ELISA kit **(D)**. Three independent experiments were performed in duplicate, and values are means ± SD for all four experiments. *P<0.05, **P<0.01. The *P* value was calculated using Student’s t-tests.

To understand the pathogenesis of the PEDV infection in porcine intestinal epithelial cells, we infected IPEC-J2 cells with mock or PEDV strain CV777 at an MOI of 1. At different time points post infection, we monitored the mRNA level for the cytokines TNF-α, IL-8, IL-12 and IL-18 by qRT-PCR as described previously (Guo et al., 2014). As shown in **Figure 1A**, IL-12 and IL-18 transcripts were maintained relatively low level in PEDV CV777-infected cells; in contrast, infection of IPEC-J2 with PEDV for 48 h and 72 h induced a sharp increase of TNF-α and IL-8 transcripts. Therefore, we evaluated the protein level of these two inflammatory factors after IPEC-J2 exposed to PEDV by ELISA. To minimize the transfer of infectious virus, the cell supernatant was removed before the cells were plated in fresh media. As shown in **Figure 1B**, the secreted IL-8 protein was gradually increased upon PEDV infection in the course of time in contrast to mock treated cells. However, although mRNA level of TNF-α was remarkably upregulated, the production of TNF-α was only detectable at 24 h post-infection (hpi) at low level (20 pg/mL, data not shown), which may be due to the TNF-α production mainly from activated immune cell types, such as macrophages (Locksley et al., 2001; Stow et al., 2009).

Vero E6 cell is a very common cell line used for growing coronaviruses, thus the expression of IL-8 in Vero E6 cells infected with PEDV was also evaluated. It is shown that significant up-regulation of IL-8 at the transcriptional levels was observed by qRT-PCR analysis at 24 to 72 h post infection (**Figure 1C**). ELISA was also used to quantify IL-8 secretion, and the data showed that cells infected with PEDV increased IL-8 production over times in comparison to mock treated cells (**Figure 1D**). These findings indicate Vero E6 cells can be used as a model cell line to evaluate the host cell response upon PEDV infection.

### 3.2 PEDV induces IL-8 production *in vivo*

**Figure 2:**
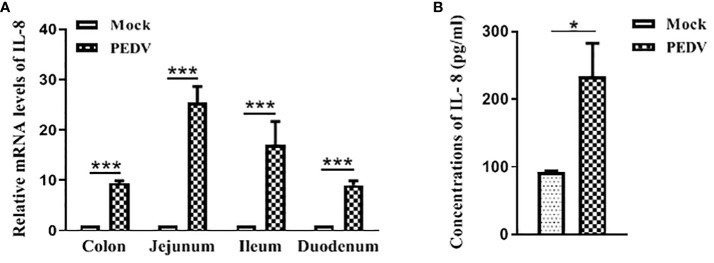
PEDV infection induces IL-8 upregulation *in vivo*. **(A)** Pigs were infected intranasally with PEDV (2 ml of 10 ^6.0^ TCID_50_/mL). Samples were collected at 2 days post infection. IL-8 mRNA was quantified by qRT-PCR. And results were normalized to GAPDH and expressed as fold induction over samples from uninfected pigs. **(B)** The concentrations of IL-8 in serums collected from piglets post-challenge were detected via ELISA assay. The data are representative of three independent experiments (the means ± SD). *P<0.05, ***P<0.001. The *P* value was calculated using Student’s t-tests.

To investigate whether PEDV has the ability to induce IL-8 production *in vivo*, SPF pigs were orally infected with PEDV strain, and samples were collected at 2 days post infection for IL-8 analysis. The intestinal tissues collected from these piglets were analyzed by qRT-PCR for IL-8 analysis. Our results showed that IL-8 mRNA expression was significantly induced in intestinal tissues from PEDV-infected piglets (**Figure 2A**). Compared with the uninfected piglets, serum levels of IL-8 were significantly up-regulated in PEDV-infected piglets (**Figure 2B**). Collectively, these data indicate that PEDV infection remarkably induces IL-8 production both *in vivo* and *in vitro*.

### 3.3 PEDV up-regulates IL-8 expression by activating NF-κB signaling

**Figure 3:**
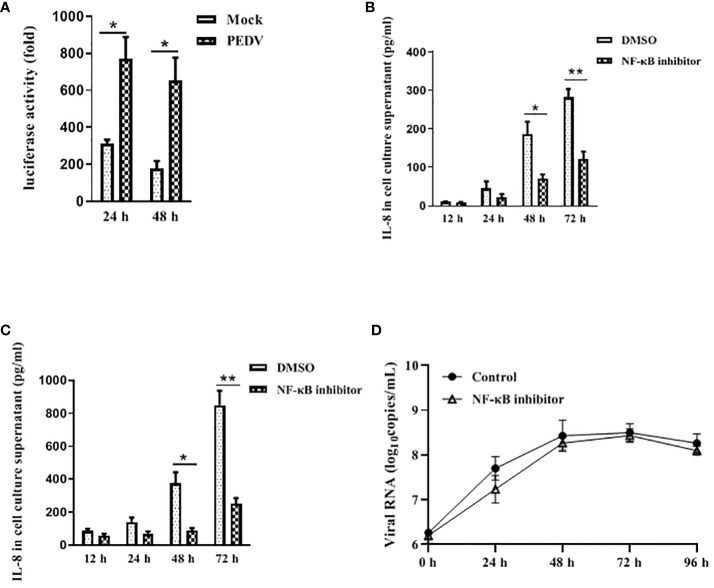
NF-κB is involved in IL-8 secretion upon PEDV infection. **(A)** PEDV infection induces NF-κB activity. Vero E6 cells were transiently transfected with the pNF-κB-Luc vector for 24 h, and followed by infection with PEDV CV777 or mock control for 24 h and 48 h. Cells were lysed, and luciferase activity was detected by chemiluminescence was measures as counts/second. **(B)** NF-κB activity is involved in the IL-8 production in Vero E6 cells upon PEDV infection. Vero E6 cells were treated with DMSO (carrier) or Bay 11–7082 (10 μM) for 1 h. Cells were then washed and infected with mock or PEDV CV777 at an MOI of 1. Supernatants were collected at different time points as indicated and assayed for the production of IL-8. **(C)** NF-κB is involved in the IL-8 production in IPEC-J2 cells during PEDV infection. The treatment procedure in IPEC-J2 is similar to Vero E6 cells, and cell media supernatants were assessed the IL-8 production by ELISA. **(D)** NF-κB activity does not affect viral replication. Vero E6 cells were treated with DMSO (carrier) or Bay 11–7082 (10 μM) for 1 h, and followed by the infection with mock or PEDV CV777 at an MOI of 1. Production of PEDV progeny was quantified by qRT-PCR using the primers specific to PEDV E gene. The data from three independent experiments are expressed as mean ± SD. *P<0.05, **P<0.01. The *P* value was calculated using Student’s t-tests.

NF-κB is an important transcription factor and plays a vital role in regulating the expression of multiple pro-inflammatory cytokines and chemokines (Roghani et al., 2023). Therefore, we initially determined whether PEDV-infected cell modulates NF-κB activity by using luciferase report system as described previously (Balasubramanian et al., 2003). Vero E6 cells were transfected with pNF-κB-Luc followed by infection with mock or PEDV. As is shown in **Figure 3A**, at 24 h and 48 h post infection, PEDV infection significantly stimulated NF-κB activation compared to the mock infection, indicating NF-κB activity is induced upon PEDV infection.

To verify whether NF-κB activation leads to the production of pro-inflammatory cytokine IL-8 in PEDV-infected cells, Vero E6 was infected with PEDV in the presence of NF-κB inhibitor Bay 11–7082 at the concentration of 10 μM. Cell supernatants were collected for IL-8 estimation at the 12 h, 24 h, 48 h and 72 h post infection. We observed that PEDV-induced IL-8 production was significantly reduced in the presence of the NF-κB inhibitor at all incubation times (**Figure 3B**). Similarly, a significant reduction of IL-8 secretion in PEDV-infected IPEC-J2 was observed when incubating cells with the NF-κB inhibitor (**Figure 3C**), indicating that the NF-κB activity is involved in the PEDV infection-induced IL-8 production.

It has been reported that activated NF-κB confers an antiviral response against cytoplasmic RNA viruses (respiratory syncytial virus and human parainfluenza virus type 3) (Bose et al., 2003). Therefore, to determine whether the NF-κB activity has effect on the PEDV replication, we used NF-κB inhibition assay to evaluate the viral replication. As shown in **Figure 3D**, although virus copy numbers were slightly decreased at 24 h post infection, at all the time points virus replication is not significantly affected in the presence of Bay 11–7082. Our data suggest that NF-κB activity has no obvious effect on PEDV replication.

### 3.4 PEDV E and M protein up-regulate IL-8 expression

**Figure 4:**
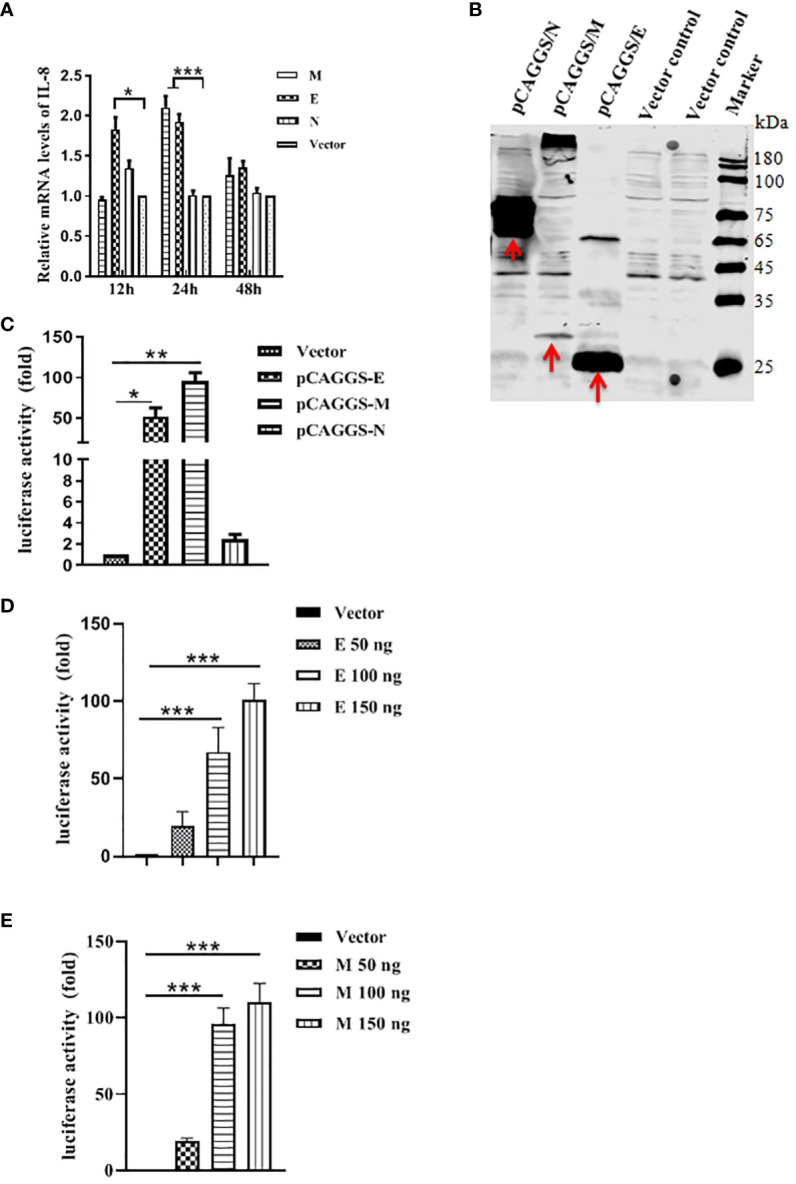
PEDV structural proteins can induce IL-8 production. **(A)** PEDV E and M proteins can induce IL-8 production. Vero E6 cells were transfected with pCAGGS/M, pCAGGS/N, pCAGGS/E or pCAGGS vector for 12 h, 24 h, and 48 h, followed by qRT-PCR to examine the IL-8 mRNA level. **(B)** Detection of PEDV M, N and E proteins expression in Vero E6 cells. Cells were transfected with above four plasmids for 24 h, and cell lysates were subjected to reducing SDS-PAGE prior to immunoblotting with antibodies to Flag. **(C)** Vero E6 cells were co-transfected with porcine IL-8 promoter plasmid, together with pRL-TK, and pCAGGS/M, pCAGGS/N, pCAGGS/E or pCAGGS vector as negative control. Cells were harvested at 30 h post-infection and assayed for luciferase activity. **(D, E)** Vero E6 cells were co-transfected with porcine IL-8 promoter plasmid, pRL-TK, and the indicated amounts of E or M expression plasmids, Total amounts of transfected DNA were kept equal by adding empty vector. Cells were harvested at 30 h after transfection and assayed for luciferase activity. The results are representative of three independent experiments (the means ± SD). *P<0.05, **P<0.01, ***P<0.001. The *P* value was calculated using Student’s t-tests.

Having found that PEDV infection is indeed induced NF-κB-mediated IL-8 production, we next sought to identify which structural protein is involved in the PEDV infection-induced production of IL-8. M, N and E genes of PEDV were cloned into pCAGGS vector and transfected into Vero E6. Each viral protein was expressed as a fusion protein with Flag tag. At different time points, cells were collected to evaluate IL-8 mRNA level by qRT-PCR. We observed a significant increase of IL-8 mRNA level when the cells treated with pCAGGS/M at 24 h or pCAGGS/E at 12 h and 24 h but not with pCAGGS/N (**Figure 4A**). We also confirmed the expression of these three proteins in Vero E6 by western blot analysis of cell lysates from transfected cells. As shown in **Figure 4B**, it revealed that specific M, N and E protein presented in pCAGGS/M-, pCAGGS/N-, and pCAGGS/E-transfected cells. Collectively, our data indicate that M and E proteins of PEDV are involved in the IL-8 secretion during PEDV infection, suggesting that PEDV replication is required for IL-8 production. To further determine that PEDV E or M protein could activate the IL-8 promoter to upregulate the mRNA expression levels of the IL-8 genes. Vero E6 cells were co-transfected with IL-8 promoter plasmid, pRL-TK and pCAGGS-E/M-HA, respectively, or along with pCAGGS-HA. At 30 h post-transfection, the luciferase activity was detected. The results indicated that E or M significantly enhanced IL-8 promoter activity (**Figure 4C**). To further validate this, Vero E6 cells were transfected with IL-8 promoter plasmid, along with pRL-TK and different amounts of the E or M expression plasmid, and luciferase activity was measured at 30 h post-transfection. The data clearly showed that overexpression of E or M was responsible for the activation of the IL-8 promoter activity in a dose dependent manner (**Figures 4D, E**).

## 4 Discussion

The inflammasome pathway is a critical early response mechanism of the host that detects pathogens, initiates the production of inflammatory cytokines, and recruits effector cells to the infection site. Many studies have confirmed that virus infection can imbalance the expression of cytokines and lead to further damage in the organism (Sallenave and Guillot, 2020; Karki and Kanneganti, 2022). As an alphacoronavirus, PEDV infections caused profound atrophic enteritis that leads to severe diarrhea and dehydration, and infiltration of inflammatory cells is seen in the lamina propria (Zhang et al., 2021). One of the important inflammatory factors is IL-8, which can mediate the recruitment of inflammatory cell to play their role on clearing the entered pathogens (Gustine and Jones, 2021; Zhu et al., 2021). Zhang et al. found that PDCoV infection causes the expression of pro-inflammatory cytokine IL-8 in serum and lead to further tissues damage of the organisms (Zhang et al., 2021). Given the important significance of IL-8 in the pathology of virus infection, we dissected the underlying mechanism of IL-8 expression under PEDV infection. In this study, we investigated how PEDV induced IL-8 production. We showed that PEDV induced IL-8 production both *in vitro* and *in vivo*.

NF-κB is a transcription factor that plays central roles in virus infection, inflammation and immune responses. The activation of NF-κB could induce the transcription of pro-inflammatory cytokine and chemokine genes (Caamano and Hunter, 2002; Li and Verma, 2002). In most unstimulated cells, NF-κB dimmers (mostly p65/p50 dimers) are localized in the cytoplasma as a complex with the IκB proteins. Upon stimulation, IκB is ubiquitinated and subsequently degradated, thus the releasing NF-κB subunits translocate to the nucleus and induce transcription of target genes (Perkins, 2007). Previous studies have confirmed that African swine fever virus or SARS-CoV-2 infection activates the NF-κB signaling pathway and up-regulates the expression of IL-1β and IL-8 (Manik and Singh, 2021; Gao et al., 2022). In the present study, the NF-κB activity is involved in the PEDV infection-induced IL-8 production. Xu et al. also found that PEDV E protein induced ER stress and significantly activated NF-κB which consequently caused the promotion of IL-8 expression (Xu et al., 2013). Recently, PEDV ORF3 inhibits IL-6 and IL-8 productions by blocking NF-κB p65 activation (Wu et al., 2020). However, the mechanisms underlying the transcriptional regulation of PEDV-induced IL-8 production is still unclear. In our study, we demonstrated that PEDV E and M protein induced IL-8 expression. However, how the viral structural proteins affect IL-8 upregulation is unclear, further work needs to conclusively elucidate this issue.

In the present study, we demonstrate for the first time that PEDV infection induces the upregulation of transcription of IL-8 and the secretion of IL-8 protein. We also found that NF-κB pathway is involved in the production of IL-8 upon PEDV infection. Moreover, the cells expressing PEDV M or E protein cause high expression of IL-8. Our findings have potentially important implications for understanding the molecular mechanisms of pathogenesis for this economically important porcine disease.

## Data availability statement

The original contributions presented in the study are included in the article/**Supplementary Material**. Further inquiries can be directed to the corresponding authors.

## Ethics statement

The animal study was approved by Harbin Veterinary Research Institute(SYXK-2018-032). The study was conducted in accordance with the local legislation and institutional requirements.

## Author contributions

YW: Writing – original draft. YWa: Writing – original draft. XW: Writing – original draft. ML: Writing – review & editing. HY: Writing – review & editing. HS: Writing – review & editing. DS: Writing – review & editing. JC: Writing – review & editing. LG: Writing – review & editing. LF: Writing – review & editing.
