# Supplementary figures and images for: Elevation of IL-8 secretion induced by PEDV infection via NF-κB signaling pathway
Source: Front Cell Infect Microbiol. 2024 Jul 22;14:1422560. doi: 10.3389/fcimb.2024.1422560 (PMC11298435; doi:10.3389/fcimb.2024.1422560)

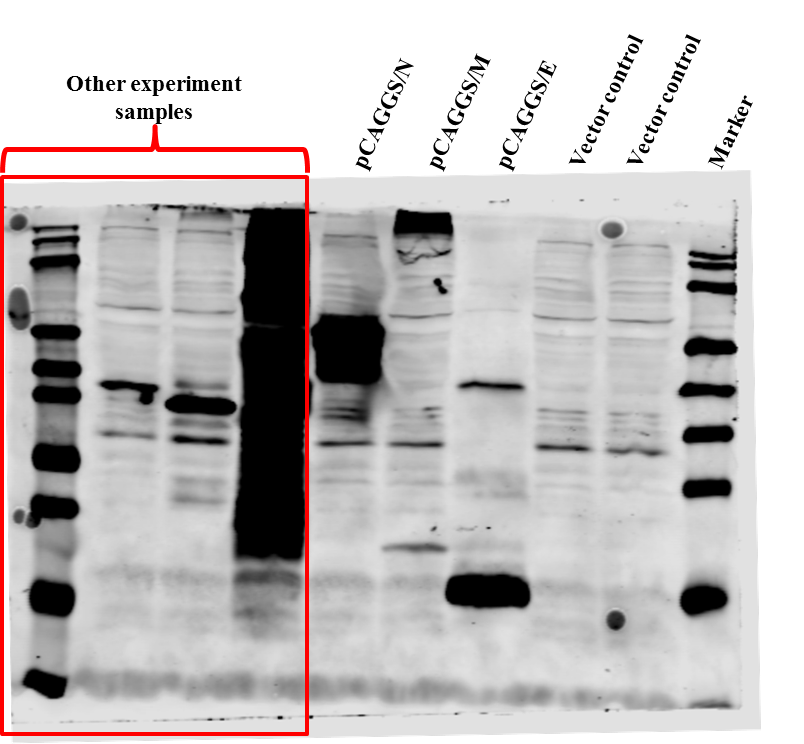

Supplement: Supplementary file 2 [file Image_1.tif]
